# Barriers to the uptake of cervical cancer screening methods in Low- and Middle-Income Countries (LMICs) – a scoping review using framework thematic analysis

**DOI:** 10.1186/s12905-026-04764-w

**Published:** 2026-08-11

**Authors:** Momina Zulfeen, Debra Howell, Omara Dogar

**Affiliations:** 1 Department of Obstetrics and Gynaecology, Sheffield Teaching Hospitals NHS Foundation Trust, Sheffield, UK; 2 Department of Health Sciences, University of York, York, YO105DD UK

**Keywords:** Qualitative research, cervical cancer screening, qualitative evidence review, low- and middle-income countries, health behaviour

## Abstract

**Background:**

Cervical cancer remains a major cause of preventable death among women in low- and middle-income countries (LMICs). Despite the availability of effective screening, uptake remains low. Existing reviews have not fully captured the socio-cultural and behavioural barriers influencing women’s participation. This scoping review explores factors influencing the uptake of cervical cancer screening in women in LMICs.

**Method:**

Qualitative studies exploring barriers and facilitators related to cervical cancer screening uptake were identified through a search of MEDLINE and Embase from January 2014 to December 2022. Qualitative data were extracted and analysed using framework thematic analysis guided by the Theoretical Domains Framework, and inductively coded to generate overarching themes.

**Results:**

A total of 17 studies were included in the review. Knowledge, beliefs about consequences, social influences, social roles and emotions were identified as the key domains of barriers affecting screening uptake. Women in low- and middle-income countries held cultural values that were deeply important to them, which were closely intertwined with health education. Discrepancies between the perspectives of healthcare professionals and women’s cultural values contributed to a heightened mistrust of the healthcare system.

**Conclusion:**

Lack of awareness about cervical cancer and misconceptions about its causes were prevalent, particularly in rural areas. Stigma, societal expectations, and gender inequalities further exacerbated the reluctance to access screening services, while procedural fears and distrust in healthcare providers deepened the problem.

**Trial registration:**

PROSPERO registration: CRD42023402109.

**Supplementary Information:**

The online version contains supplementary material available at https://doi.org/10.1186/s12905-026-04764-w.

## What is already known on this topic

- Despite cervical cancer being largely preventable through vaccination and screening, it remains a leading cause of cancer-related deaths among women in LMICs.
- Research shows that low screening rates in LMICs are often linked to knowledge gaps, stigma, and limited healthcare. However, most reviews use quantitative methods or focus on specific areas like Sub-Saharan Africa, frequently missing the nuanced socio-cultural and emotional factors influencing women’s choices about cervical cancer screening. As a result, many barriers in diverse LMIC settings are not fully addressed.

### What this study adds

- This scoping review provides an in-depth exploration of barriers to cervical cancer screening across LMICs, drawing together the perspectives of women and healthcare providers through a framework-based thematic analysis using the Theoretical Domains Framework.
- The review highlights how cultural values, emotional fears, stigma, and mistrust in healthcare systems intersect to shape women’s screening decisions.
- It reveals that misconceptions about causation, fear of cancer and its consequences, gendered expectations, and prior negative healthcare experiences are deeply interlinked barriers that go beyond mere lack of awareness.
- By identifying the emotional, social, and structural roots of these barriers, this study offers a more comprehensive understanding of why screening uptake remains persistently low.

### How this study might affect research, practice or policy

- The findings underscore the need for cervical cancer screening interventions that are culturally sensitive, community-engaged, and trust-building rather than solely informational.
- Health promotion strategies should address fear, stigma, and gender dynamics alongside education, ensuring respectful care and the availability of female providers where culturally appropriate.
- For policymakers, integrating screening into primary care and community-based settings, supported by peer educators and local leaders, could improve accessibility and acceptance.
- Future research and implementation efforts should focus on context-specific, scalable interventions that tackle the socio-cultural determinants of screening behaviour.

## Background

Cervical cancer is one of the few preventable cancers which can be cured if detected early. Cervical cancer is known to be caused by a virus called HPV (Human Papillomavirus), which may lead to precancerous changes that can progress to cancer over decades, thus providing a window for early detection and treatment. Cervical cancer is also preventable through HPV vaccination; however, implementation of vaccination programmes remains challenging in many Low- and Middle-Income Countries (LMICs).

Despite clinically effective secondary and primary prevention strategies, cervical cancer remains the most common type of cancer in LMICs, where 80% of the cases occur [1]. Globally, one woman dies from cervical cancer every two minutes [2]. The burden of cervical cancer is disproportionately high in LMICs, accounting for over 85% of deaths. Even though cervical cancer is largely curable, poor coverage of secondary prevention strategies is a significant barrier in reducing cancer-related mortality and morbidity [2].

Primary and secondary prevention strategies rely on HPV vaccination and early detection through cervical screening, respectively [3]. While HPV vaccination has been widely implemented in high-income countries, financial and infrastructure constraints have limited its uptake in many LMICs. Recognising these challenges, the World Health Organisation (WHO) has emphasised improving access to secondary prevention strategies in LMICs [4, 5].

The prolonged latent phase of cervical cancer allows opportunities for early detection and treatment [6]. Although cytology-based screening (Pap smear) and HPV testing have substantially reduced cervical cancer incidence and mortality in high-income countries, their widespread implementation in LMICs is often limited by resource constraints. Consequently, alternative approaches such as visual inspection with acetic acid (VIA) have been introduced, although screening uptake remains low [7, 8]. Several barriers, such as a lack of awareness and social stigma, have been recognised but remain to be overcome. For example, a review of knowledge, attitudes and practice on cervical cancer screening in India revealed that only 5% of eligible women had undergone screening compared to 84% in high-income countries [8]. As the efficiency of a screening programme is contingent on uptake, recognising barriers is vital to overcome challenges and achieve the intended outcomes [9, 10]. However, most evidence on barriers to screening is based on quantitative studies [11, 12], which, while valuable for measuring prevalence and associations, often fail to capture the nuanced, context-specific factors that influence screening behaviour. This creates a gap in understanding the underlying social, cultural, and psychological barriers that impact uptake.

In the context of complex behaviours with complex relations between patients, providers and programme implementation, qualitative research can promote an understanding of “why promising clinical interventions do not always work in the real world” [13]. A qualitative evidence synthesis from LMICs in Africa [14] identified four major themes, as barriers to screening, using thematic analysis. However, this study focused solely on barriers in Sub-Saharan Africa and did not assess interventions to reduce the barriers [14]. To our knowledge, while prior qualitative reviews have focused on specific regions such as Sub-Saharan Africa, there remains a lack of broader global syntheses encompassing diverse low- and middle-income countries through a structured theoretical framework.

This scoping review aimed to answer the research question: What are the key barriers and facilitators to, and how do they affect, the uptake of cervical cancer screening among women in LMICs?

## Methods

This review was conducted within the paradigm of pragmatism, acknowledging that women's screening behaviours are shaped by complex socio-cultural and contextual meanings [15].

Our scoping review uses framework thematic analysis using a preexisting/a priori framework. The suggested theoretical approaches/frameworks to implementation science vary depending on the objectives being accomplished. For example, when understanding ‘what influences intervention outcomes’, Nilsen et al. [16] recommend using “Determinant Frameworks”. While models like the Health Belief Model (HBM) [17] or Theory of Planned Behavior (TPB) [18] are commonly used in screening research, they primarily focus on individual cognitive factors such as perceived risk or personal attitude. TDF was specifically selected because it synthesises 33 theories and 128 constructs into 14 comprehensive domains [19]. This broad scope makes it uniquely equipped to capture not only individual cognitive and emotional factors (like fear or knowledge), but also social norms, environmental context, resource barriers, and healthcare system interactions without restricting the analysis to a single theoretical lens [19]. The framework analysis also aligns with this study's epistemological paradigm stance of pragmatism [20].

Given that cervical cancer screening uptake is influenced by cognitive, emotional, social, cultural, and healthcare system factors, the TDF provided a suitable theory-informed framework for understanding these complex behavioural determinants. We therefore conducted a framework thematic analysis using the Theoretical Domains Framework (TDF), whereby qualitative data were deductively coded into the relevant TDF domains, followed by inductive coding to generate themes through an iterative analytical process [19].

The review followed guidance from the Cochrane Qualitative and Implementation Methods Group and adhered to best practices for qualitative synthesis [21]. A purposive search strategy was developed and conducted on the following databases: MEDLINE, Embase, PsycINFO, Social Policy and Practice from January 2014 to December 2022. (Appendix 1- Search Strategy). A starting point of 2014 was used because the WHO Comprehensive Cervical Cancer Control guidelines, published in 2014, marked a significant development in cervical cancer prevention and screening policy [22]. Restricting the review to this period was intended to ensure that findings reflected contemporary screening programmes and implementation contexts. Furthermore, previous evidence suggested that the majority of relevant qualitative studies had been published after 2014. Consistent with qualitative review methodology, the aim was to achieve conceptual richness rather than an exhaustive historical review [23].

Unlike quantitative systematic reviews aimed at statistical pooling, qualitative scoping reviews prioritize conceptual depth and broad contextual representation, ensuring the mapped evidence remains sensitive to qualitative paradigms [24]. Accordingly, the search strategy sought to maximise coverage of LMICs and conceptual diversity. The SPIDER (Sample, Phenomenon of Interest, Design, Evaluation and Research type) was used to design the search, and is an adaption of the traditional PICO (Population Intervention, Control and Outcomes) [25] for quantitative research (Table 1 and Appendix 1). Identifying qualitative research can be challenging due to the absence of standardised search terms. Therefore to maximise the identification of “qualitative studies”, the search strategy included keywords suggested by Evans et al. [26] to improve the yield of qualitative studies.

**Table 1:**
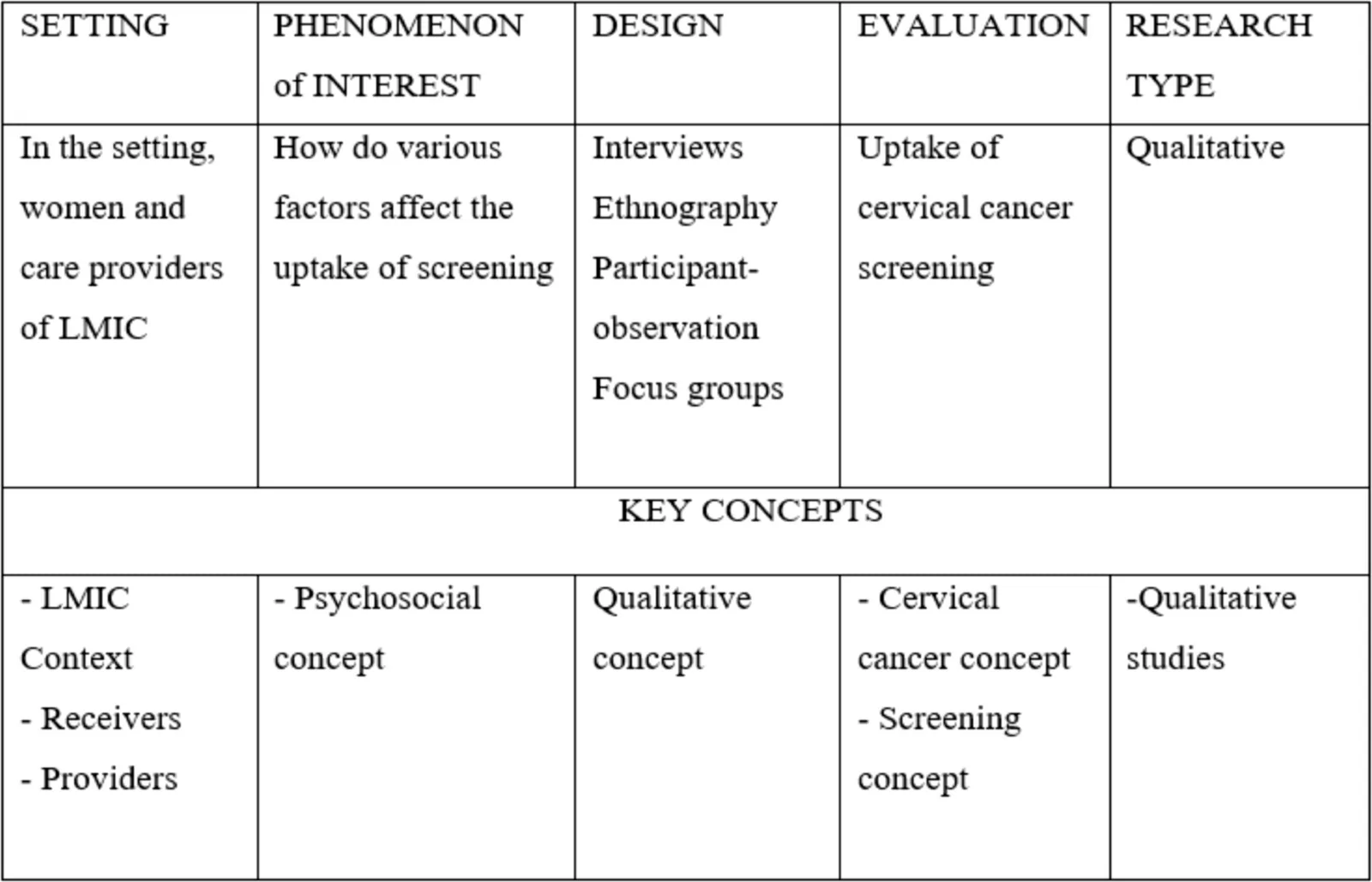
SPIDER concept for search strategy

Titles and abstracts were screened, followed by full-text review of potentially eligible articles according to the predefined eligibility criteria (Table 2). Studies were selected to include those from which an interpretive explanation could be derived, consistent with a purposive rather than exhaustive approach [27]. Uncertainties regarding study eligibility were resolved through discussion with dissertation.

**Table 2:** Eligibility criteria

Inclusion criteria:
• Studies that explore barriers and facilitators to the uptake of cervical cancer screening in LMICs
• Design—Qualitative study designs including ethnographic research, phenomenological research, participatory research, focus groups, interviews, case studies, process evaluations and mixed methods research. Where mixed methods are used, studies will be included if qualitative data can be extracted
• Participants – Women eligible for cervical cancer screening in LMICs, as well as male partners, healthcare providers, community leaders, or other stakeholders where their perspectives contributed to understanding women's screening uptake
Exclusion criteria:
• Studies that explored factors in immigrant populations in developed countries
• Studies exclusively exploring provider perspectives without reference to barriers or facilitators affecting women's cervical cancer screening uptake
• Studies exploring factors related to HPV vaccination alone
• Studies exploring factors related to follow-up screening alone
• Studies that did not meet inclusion criteria were excluded
• Special populations including health care providers as patients, immigrant women and women with HIV, mental disabilities and physical impairment
• Adolescents or < 18 years
• Studies before 2014, excluding 2014
• Unpublished articles
• Conference abstracts without full text
• Full texts that are not retrievable

Barriers and facilitators identified in the studies were iteratively coded ‘line by line’ by the author (MZ) to the 14 TDF domains and relevant themes within Nvivo [28], a software tool to manage and code text, and facilitate data analysis [29]. As this was a scoping review, quality assessment was not conducted on the included studies.

We conducted a framework thematic analysis using the TDF (Theoretical Domains Framework), in which qualitative data were extracted and deductively coded into predefined TDF domains, followed by inductive development of themes within these domains via an iterative process [19, 30].

Overlapping concepts were coded into each relevant category, and “Memos” were used to record analytical decisions. Coded references were retrieved and reviewed for correctness, and themes were refined within the TDF domains, although extracts could be coded to multiple domains (Table 4). Coding was undertaken by a single reviewer, and uncertain domain or theme assignments were reviewed with the second reviewer. Formal double-coding or inter-coder reliability assessment was not undertaken.

## Quality of evidence

A formal quality assessment of individual studies was omitted, consistent with qualitative review approaches prioritising conceptual diversity. However, by adhering to Cochrane Qualitative and Implementation Methods Group guidance on qualitative design and maintaining transparency and rigour at each review stage, the author attempted to produce reliable findings in lieu of this study's ‘pragmatic’ stance [31].

## Results

**Fig. 1:**
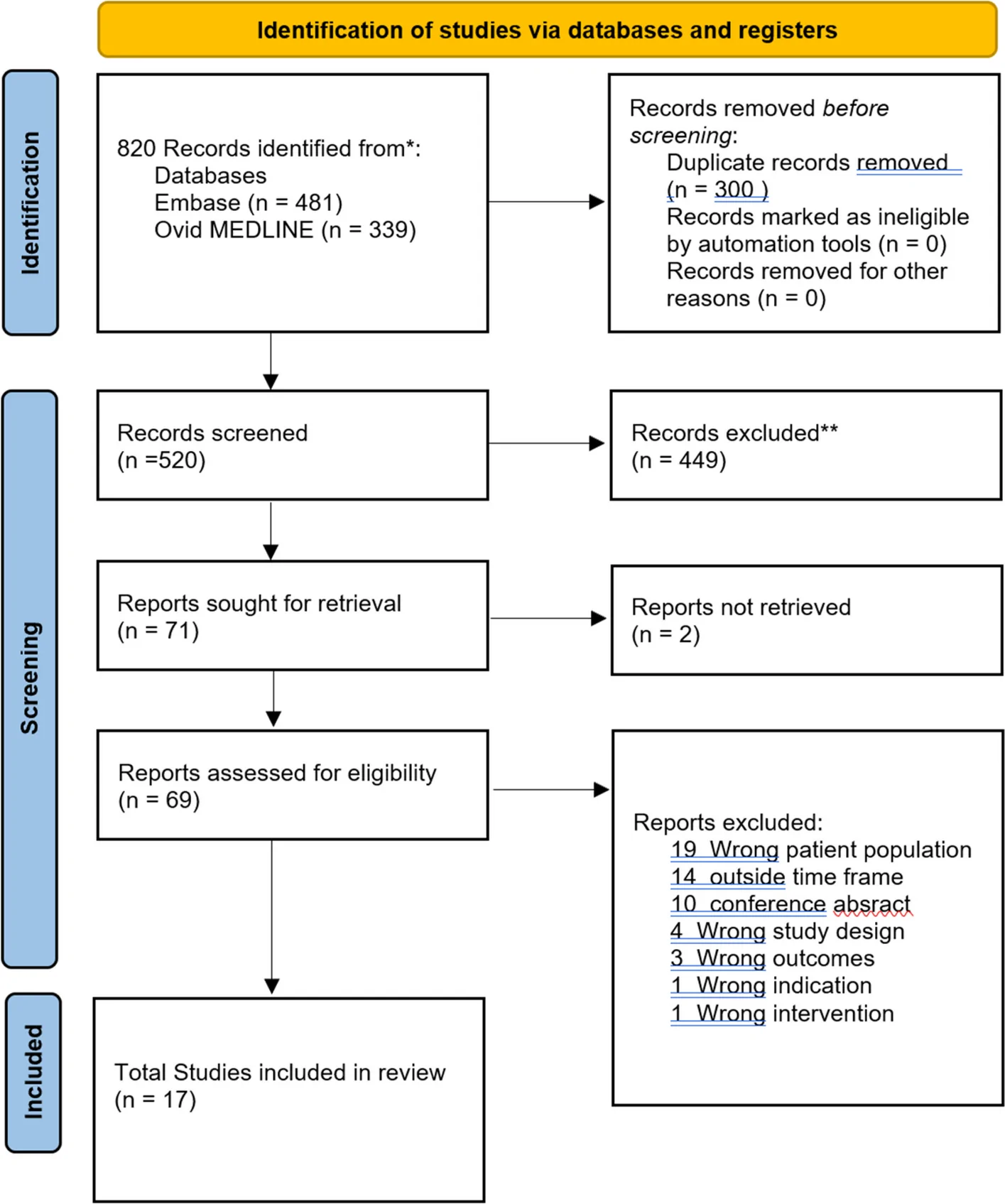
PRISMA Flowchart. (From: Page MJ, McKenzie JE, Bossuyt PM, Boutron I, Hoffmann TC, Mulrow CD, et al. The PRISMA 2020 statement: an updated guideline for reporting systematic reviews. BMJ 2021;372:n71. https://doi.org/10.1136/bmj.n71)

The search identified 820 records, comprising 481 records from Embase and 339 records from Ovid MEDLINE. After 300 duplicates were removed, 520 records underwent title and abstract screening. 69 were selected for full-text review, and 17 studies were eligible for the review, with methods mainly including focus group discussions and interviews (Fig. 1- PRISMA flowchart).

## Description of studies

Participants were mainly women or mixed gender, except for one study where all participants were male [32]. Table 3 shows the characteristics of the included studies.

**Table 3:** Characteristics of included studies

Study	Aims	Methods	Participants	Country	Notes
Modibbo 2016 [33]	To explore knowledge, attitudes and beliefs about cervical cancer prevention	Focus groups	27 Christian / 22 Muslim women aged 18 + with no cancer diagnosis	Nigeria	
Khazaee-Pool 2018 [34]	To explore perceptions / experiences of cervical cancer prevention	Semi-structured in-depth interviews/ focus groups	27 women, aged 20–60 years, with no previous history of cervical cancer symptoms or diagnosis	Iran	
Ali 2021 [35]	To explore lived experiences in doctors providing cervical cancer screening	Semi-structured interviews using applied grounded theory	12 gynaecologists and one general practitioner (GP) working in two main hospitals	Iraq	
Moukam 2021 [36]	To understand barriers affecting decisions to take part in screening	Focus groups	43 Women aged 30–49 years, and male partners	Cameroon	
Leibermann 2020 [37]	To explore knowledge and attitudes to human papillomavirus (HPV), cervical cancer, screening, and barriers and facilitators to early detection	Focus groups	64 Parents and grandparents of school age children in public/private schools, workplaces and community settings	Dominican Republic	Part of a larger study on barriers and facilitators to HPV vaccine
Marashi 2021 [38]	To investigate barriers to Pap smear	Focus groups and individual semi-structured interviews	32 Women, health staff, obstetricians, and gynaecologists aged 26 – 40 years	Iran	
Teng 2014 [39]	To define and understand embarrassment and its role in cervical cancer screening and self-collected HPV DNA testing	Semi structured one-to-one interviews and focus groups	6 Key-informant health workers and 16 local women aged 30–69 years	Uganda	Specifically explores the impact of embarrassment on cervical screening
Binka 2019 [40]	To explore barriers to cervical cancer screening uptake and treatment	In-depth interviews and focus groups	15 Patients with cervical cancer attending clinic; women aged 30–65 years and never screened	Ghana	
Tapera 2019 [41]	To investigate knowledge, attitudes, beliefs and practices towards cervical cancer prevention and treatment	Interviews	134 Key informants aged 18 – 45 years: with cervical cancer, health workers and others involved in cancer control programmes	Zimbabwe	Sequential explanatory mixed methods study design
Darj 2019 [42]	To explore perceptions of cervical cancer screening, and barriers and facilitators to participation	Focus groups	72 married women, fluent in Nepali, aged 25–60 years	Nepal	Content analysis
Nyblade 2017 [43]	To assess perceptions of cervical cancer, screening, and gynaecological examinations, and explore barriers and facilitators to screening/seeking gynaecological services	In-depth interviews and focus groups	147 women, husbands, healthcare providers and community leaders aged 30—60 years	India	Two qualitative studies; one cervical cancer, the other breast cancer
Lunsford 2017 [44]	To explore perceptions about barriers and benefits of cervical cancer screening	Focus groups	60 women aged 25–49 years; 40 male partners in 10 focus groups (6 female, 4 male), in rural and urban settings	Kenya	
Rasul 2016 [45]	To explore perceived barriers to cervical cancer screening	Semi-structured in-depth interviews	Kurdish women who had had, or refused a Pap smear	Iraq	
Mahalaxmi 2020 [46]	To explore barriers and enablers to breast and cervical cancer screening uptake among women	In-depth interviews	19 women survivors, community members with or without symptoms, service providers, professionals in oncology and community medicine aged 30 – 86 years	India	
D’Souza 2020 [47]	To identify barriers preventing cervical cancer screening uptake	Focus groups and interviews	45 women aged 30–59 years; Programme managers/health care staff implementing screening	India	
Asl 2020 [48]	To explore awareness of cervical cancer testing and role of knowledge, perceived risk, and cues to action	Focus groups and in-depth interviews	79 women, married, aged 25–65, and Farsi-speaking	Iran	
Fentie 2020 [49]	To identify factors associated with screening uptake, VIA (visual inspection with acetic acid) positivity and predictors	In-depth interviews	15 women aged 26 to 40 years screened and not screened	Ethiopia	Concurrent mixed methods qualitative interviews and cross-sectional
Okedo-Alex 2020 [32]	To assess knowledge and attitudes towards cervical cancer screening	Focus groups	16 married men in a rural community	Nigeria	Mixed methods study
Hammerli 2022 [50]	To explore factors affecting community healthcare worker motivation in a cervical cancer screening program	In-depth interviews with community healthcare workers	Four women, seven men aged 21–77 years	Dschang, Cameroon	

## Analysis and findings

### Deductive analysis

Using NVivo, code barriers were identified and grouped into relevant TDF domains. For example, the data excerpt ‘Participants among the Abuja Muslim women gave the need for spousal support and permission before the screening, need for a female doctor to be present during screening procedure as reasons for reluctance to screening’ was coded into both “social influences” (interpersonal influences like spousal support) and “social, professional role and identity” (identification with Muslim culture).

TDF domains are defined in Appendix 2.

### Inductive analysis

A narrative analysis of the six most frequently identified TDF domains is presented below, with themes developed inductively from the data within each domain. The other less commonly identified domains are described in Table 4.

**Table 4:** Inductive analysis of TDF domains

TDF domain	Identified codes (n)	Barriers-Patient related	Underying themes	Illustrative quotations (abridged)
Behavioural regulation	5	Lack of motivation Lack of a recall system	Difficulty changing a previous routine activity	*“Humans are naturally lazy, and if there is nothing to force them to do this test, no one will do it.”*
Beliefs about capabilities	8	Long waiting times Lost work	Lack of perceived ability to go through screening	*“it is difficult, time-consuming, and exhausting.”*
Optimism	7	Lack of counselling post diagnosis Lack of awareness	Pessimistic to nihilistic attitude	*“Life is finished nothing is there after getting cancer…”*
Beliefs about consequences	85	Lack of awareness Unpleasant experience with testing Poor data protection Discrimination at facility and societal levels	Consequences to femininity	*“Living without a uterus or genital organs is meaningless. “*
			Abnormal results	*“ What you don’t know won’t hurt you.”*
			Breach of confidentiality	*“secret would come out.”*
			Discrimination	*“ They wouldn’t take her for work at all.”*
			Pain or discomfort	*“… I think the doctors should be more gentle.”*
Emotion	92	Poor trust in hospitals Lack of awareness Fear and anxiety relating to cervical cancer Embarassment at screening centres	Anxiety regarding false positives and missed diagnoses	*“I can’t believe the hospital and its diagnosis. don’t really”*
			Fear of cancer	*“Being diagnosed with cervical cancer is like a death sentence.”*
			Fear of unknown	*“Women would rather not know.”*
			Fear or embarrassment with screening	*“For me, I feel shy to open that place for anybody.”*
			Denial	*“Talking about cervical cancer brings bad chance.”*
Environmental context and resources	36	Lack of facilities Poor access Health care worker shortage Referrels to farther centres Poor quality services	Convenience	*“Lack of material* *such as umbrellas or boots to help them reach women during the rainy season.”*
Goals	25	Lack of awareness of screening purposes Lack of concept of prevention of cancer	Perceptions of screening outcomes	*“For those having problems should do this test. Simply otherwise there is no need to do it.”*
			The apparent sense of health	*“… for me everything is okay.”*
Intentions	4	Lack of informed consent/motivational counselling	Lack of motivation to prevent cancer	*“I went to the doctor for a Pap test. I was so glad, and I forgot all my doubts and fears when the doctor informed me about my genital health.”(facilitator)*
Knowledge	158	Lack of awareness Misconceptions	Lack of knowledge of cervical screening	*“Screening was used to detect other uterine complications, too”*
			Lack of knowledge on cervical cancer	*“I don’t hear anyone say Cervical Cancer, it is called uterine cancer.”*
			Supernatural beliefs and misconceptions on the causes of cervical cancer	*“I think you know women have a lot of…like herbs that they insert into their vagina.. most of women there have cervical cancer”*
Memory, attention and decisional processes	11	Lack of invitations	Lack of reminders	*“In developed countries, each woman has her* *own health records. So, if someone forgets to do the test, they will contact her..”*
Reinforcement	55	Financial difficulties unaddressed Lack of supportive environment	Lack of financial incentives/ compensation	*“If I had a job, I would have paid for the cost of the test.”*
			Inability to share or discuss	*“Women explained that discussing the topic of* *screening raised questions about their modesty.”*
			Peer support or influence	*“All women endorsed peer-to-peer education and engaging local leaders as some of the best strategies to recruit women.”*
			Social support	*“If we say we want to do, they will be like ‘Why do you want to do? There is no need.”*
Skills	11	Insufficient training of HCP Unfamiliarity with self sampling	Lack of trust in community worker training	*“One of the doctors trained herself using videos from YouTube.”*
			Lack of confidence in self-sampling	*“unable to take adequate samples of good quality even with instructions.”*
Social and or professional role and identity Social influences	151	Social roles as a woman Religious influence Lack of decision making power Social stigma related to cancer	Prejudiced social status as women	*“they are scared that if they have disease husband may leave her and go to another lady.”*
			Religious identitiy	*“Islamically it is not good to expose yourself outside, except to your husband.” Ondo Muslim FGD participant*
			Spousal influence and financial dependence on men	*“If I as her husband does not instruct her, then she should not try it.”*
			**Barriers linked to stigma** - Association with sex - Cancer as contagious - Sense of misfortune or punishment - Social outcast	*“This is like a contagious disease, if one is diagnosed* *of cancer, we should not allow children near them.”*
		Substandard HCP behaviour Clash with local/alternative medicine	**Health care setting** - Information from HCP - Past HCP experiences - Poor trust in health facilities - Long waiting times	*“They do not want to listen to us.”* *“ Herbal medicines are better.”*
			Anti- modern medicine stance	
			Common sharing of misconceptions	

## Domain knowledge (13 studies)

Knowledge is the most frequently identified domain among the studies, with “Lack of knowledge” being the most common theme within this domain. Thirteen studies identified a lack of knowledge about cancer screening or cancer itself as a barrier [33–35, 37–39, 42, 46–51]. For example, Nigerian research reported a lack of awareness of cervical cancer:

“*None of the participants in this group previously believed that cervical cancer is becoming a significant problem or that they could get cervical cancer*” [33].

Lack of awareness was also common in this domain, presenting as false positivity. One of the perceived reasons for this was the reluctance to share information or to discuss cervical cancer [48]. Women lacked awareness regarding prevention, causation, treatment, and screening. A lack of information was often cited as the reason for such limited knowledge. Women did not think screening was required if they were healthy and monogamous. They also thought that vaginal deliveries were protective, and screening wasn’t necessary:

### Theme: misconceptions- vaginal health practice and beliefs

A theme identified within these studies, and that may be more common in developing countries, was ‘misconceptions’, for example, about vaginal health practices and supernatural beliefs: "*Among the Christian women in the Abuja FGDs, one of the most common misconceptions was that cervical cancer could result from wizardry*” [33].

Another recurring misconception was the incurability of cervical cancer and that screening only detects the presence of cancer:

*“Cervical cancer screening is only used for the detection of cancer that is already present*” [49];

*“If developing cervical cancer will be diagnosed in me, I would reject doing the treatment like chemotherapy, since the last outcome is alike and is death*” [34];

*“Nowadays, people say that if someone to gets cancer, at least let her get HIV, than getting cancer, because for HIV there is treatment, but for cancer, there is no treatment*” [39]

Screening was also thought to be needed only if symptoms appeared:

*“I do not want to perform the preventive behaviour like a Pap smear, due to the lack of any symptoms or problems*.” [34].

Misconceptions relating to causations were varied, but commonly associated with intercourse, promiscuity and unhealthy lifestyles. Lack of awareness was more common in rural than urban areas [37].

### Theme: misconceptions—supernatural beliefs

Religion and culture had a significant impact on women’s perceptions. A recurring belief was the sense of punishment, either that they were being punished by a greater power caused by wizardry or that prayers prevented cancer:

“*This disease will come only to people who commit sin*” [46];

Traditional healers played an essential role in treatments, and women believed they could also prevent the disease. Such superstitions are strong enough for women to avoid discussing cancer:

*“I don’t want to talk about cervical cancer at all … Talking about cervical cancer brings bad chance*” [34].

## Domain—beliefs about consequences (9 studies)

Beliefs about consequences were a recurring barrier across nine studies [33, 34, 36–39, 42–44]. Overlapping with the domain of emotion, negative perceptions of the outcomes of screening and cancer were prevalent. The following themes were apparent from the inductive analysis.

### Theme: consequences to femininity

Women strongly associate reproductive health with femininity:

“*I rather wish to be dead than to live without my uterus and genital organs*” [34].

### Theme: consequences of abnormal results

Women feared having to undergo further tests for abnormal results. Losing reproductive organs was considered unacceptable, and women perceived biopsies as harmful:

“*Some women perceived that before they got a biopsy/test they were healthy and after the test, they were sick, so it must have been the test that made them sick*.” [39]^,^ [52]

Other themes within this domain were the perceived consequences of a breach in confidentiality, discrimination and pain/discomfort related to the procedure. A commonly reported barrier was the fear of ‘everyone knowing’ as might occur in rural contexts:

*“There is a government hospital in the village. If they go for test there, they feel the information will leak out and everyone will come to know about their problems. Hence, they are afraid to get tested at times*.” [43]; “*The respondents said they may not go for screening if they did not trust that the health personnel would keep their information confidential*” [40].

Discomfort and discrimination at the procedure site were commonly reported across studies. Women attending clinics are often met with verbal abuse, and blaming by the health personnel. Such behaviour is perceived as discrimination and prevented women from going to screening centres:

“*I just performed a Pap smear four years ago … because I found it is very painful, I decided not to perform it anymore … I think the doctors should be more gentle … I mean, I did it in [the clinic], and the physician pulled my uterus too hard. It was intolerable … it was awful, so I do not desire to perform it once again*.” [34]; “*women with cancer would likely no longer be able to work outside the home, either because they were too weak, or because employers would discriminate against them and not allow them to work*” [43].

## Domain: emotion (9 studies)

Emotion was a commonly identified TDF domain, with fear and anxiety being reported most often [33, 34, 36–40, 50, 53].

### Theme: fear

Fear of cancer was present before and after screening. Women avoided screening due to fear of cancer and even avoided discussions about cancer. The underlying reasons included fear of: death, discrimination, marital difficulties and of losing femininity:

A male participant stated “*When a woman is going to get screened, it becomes difficult to tell her husband because her husband, upon getting this news, will think that she has already acquired cancer from someone and he could think that the wife is going out and being promiscuous. It could cause a break up in the family*” [44].

Fear of death was prevalent, and women were unaware of the treatment options; they considered the diagnosis a death sentence:

“*Women would rather not know and live with cervical cancer than find out they have it, not know what to do, and have their heart break*” [44]; “*I am scared, what will I do, if they say, I have cancer*” [46].

### Theme: embarrassment

Women from LMICs invariably perceived embarrassment during screening and preferred self-sampling approaches. However, their trust in the test results with self-sampling was low. Various factors caused embarrassment, including the gender of the healthcare worker, their religious and social identity, and shyness due to having to expose their genitals. Women preferred female test-takers to males. Interestingly, women felt that once they were aware they had a HPV diagnosis, they did not fear further tests. Furthermore, it is common practice in certain countries to perform vaginal douching, and women did not think the physical aspect of screening was a barrier in itself, but the lack of familiarity was. Unfamiliarity with staff at screening centres and in hospital environments could provoke embarrassment, rather than exposure per se.

“*I don’t think it has to do with shyness, because women they actually douche, they use herbs for all sorts of things. Even when they are pregnant, they use herbs, it is in most of cultures here, particularly in central Uganda, you use herbs to enlarge your birth canal to make sure you don’t get an episiotomy”* [39]*.*

*“Before one year, I was examined for cervical cancer by a male health care provider and I was so ashamed when he asked me to undress. It was very difficult to accept and I think this might be because of our culture which is so sensitive to such things”* [49]*.*

## Domain: reinforcement (8 studies)

A domain capturing factors and barriers influencing screening behaviour by reinforcement was identified in 8 studies [34, 36–40, 42, 44, 46]. Themes induced from the coded excerpts were: ‘Peer support or influence’, ‘Social support’, ‘Inability to share’ and ‘Financial constraints’.

Women faced significant cost constraints for screening, even when freely available, due to the cost of travel, childcare and any further investigations:

“(*there are) the additional costs of transportation, being absent from work, and having to take care of their children*” [36].

Women also believed hospitals charge for unnecessary blood and urine tests, so their trust in healthcare providers was low:

“*Getting screened is very expensive and is not a priority when I have my kids to feed or to pay for electricity. I will only get screened if I have a symptom or I have a surplus of money*” [44].

As women were often the sole breadwinners, such costs could significantly impact their lives. Irrespective of their financial status, women often had little say in discussions and decisions related to their reproductive health, as they felt this could cause issues in marital and family life:

“*The women explained that discussing the topic of screening raised questions about their modesty or trust in their partner, which made them feel uncomfortable*” [47]; ‘*Woman can’t go independently anywhere .... she has to take consent from in-laws or husband to come to the hospital*” [47].

What acted as a significant facilitator in LMICs was peer assistance and community support from local leaders:

“*If I saw that most of my relatives did the test, it would possibly affect me and I would go to do the test*.”; “*All women endorsed peer-to-peer education and engaging local leaders as some of the best strategies to recruit women*” [39].

## Domain: social influence and social roles (17 studies)

Social influences and social roles were predominant domains, found in all the included studies. Due to significant overlap, the themes were deduced together, and seven themes were identified through inductive analysis (Table 4). Two major themes are described here.

### Theme: stigma

The overlapping theme of stigma exists across the domains of emotion and social influences. Link et al. conceptualised stigma as a “powerful social process that is characterised by labelling, stereotyping, and separation, leading to status loss and discrimination, all occurring in the context of power” [54]. Strongly associated with the emotion of a woman and its interlinking with how society treats her, stigma is central to the barriers to screening [55]. Within this theme, further categorisation was possible as ‘association with sex and promiscuity’, ‘cancer as contagion’, ‘sense of misfortune’ and ‘social outcast’.

*“If we do not clean our private parts regularly, then it leads to cervical cancer. Also, keeping multiple sexual partners causes cervical cancer. (FGDC3) Yes, we have heard about cervical cancer. For that, we have to stay away from our husbands, and take medicine regularly.”* [42]

Women also feared losing the image of being a virgin before being married, and married women feared being blamed for extramarital affairs:

“*When a woman is going to get screened, it becomes difficult to tell her husband because her husband, upon getting this news, will think that she has already acquired cancer from someone and he could think that the wife is going out and being promiscuous*.”(quote by male participant) [44].

It was also common to think of cancer as a contagious disease; hence women were shunned for it:

“*This is like a contagious disease, if one is diagnosed of cancer, we should not allow children near them*” [46].

Women fear societal rejection as a result of diagnosis and being ‘found out’ outside of hospital/clinic. The fear of rejection existed both outside and within the family.

“*If the society comes to know that is all, [I] fear [that], they will isolate us*” [46].

### Theme: Barriers relating to the healthcare setting

Perceptions regarding healthcare settings and their interactions with the general population remained significant in cancer screening uptake. Subthemes under this theme included ‘information from Health care providers (HCP), ‘Past HCP experiences’ and ‘poor trust in health facilities’. Trust in health facilities was severely lacking, and women often received little information when visiting health centres. Women valued the healthcare professional’s advice and showed readiness to undertake screening on the advice of their doctors. However, women stated this was lacking and felt that the healthcare staff did not give sufficient information:

“*Doctors do not open the discussion and don’t have time … when you ask more questions, doctors get upset”* [48]*;” nurses don’t know anything about cancer*” [41].

Another significant barrier to uptake at health care centres was past experiences with HCPs. The past experiences were not limited to screening but also included other services. Women frequently narrated worrying experiences with their doctors and nurses, including inhumane conduct and discrimination:

“*The way they are treating us in the hospital, as if we are not part of the community. Even the female doctors in the hospital, before they even attend to you, they will already be abusing you from head to toe as if you don’t know anything, as if you are not part of the community*.”

“*You know others initially traumatise people. For example, the woman [referring to a female HCP] who was recording there, she [….] asks Poupoupou questions (brutally/quickly)! [laughs] [….] She stresses you out by asking the questions quickly. No, that’s not the way to do it. She needed to slow down a bit*.” [36]

Women also perceived primary health centres as unreliable and inefficient:

“*These women even refuse to attend training held in classes health center. Overall, people regard the healthcare system as an inefficient and unreliable system, and on the contrary, refer to larger cities with better healthcare facilities*.” [38]; “…*most of these women don’t come to Government hospitals because they say, that here they put PPIUCD (*PostPartum IntraUterine Contraceptive Device) *without telling them*” [47];

“*Facilities and equipment used in the hospital for my test was a barrier. I was afraid that dirty and unsterile equipment might be used*.” [45].

### Facilitators

While women expressed the need to address barriers to encourage screening uptake, studies identified specific facilitators that could improve the effectiveness of interventions. Among these, women-friendly awareness programmes targeting screening and cancer education were acceptable to women. Women also welcomed television and media educational programmes; however, they thought these to be too short [49]. Respondents universally believed in healthy living, and some women associated pap tests with ‘healthy living’ [34]. While awareness that precursors of cervical cancer can be identified before the onset of symptoms was not prevalent, some women believed that ‘cancer hides’. This encouraged women to undergo pap smears:

“*It hides. I have heard that it’s not detected right away. In other words, the person who has it might not present symptoms for months and even years*.” [37].

Some respondents believed that making screening compulsory would be an effective tool and that organising government screening camps near homes could encourage screening. Low costs and financial incentives were among the other identified facilitators. Having ‘role models’ in the community who prevented cancer through screening was seen as inspirational and could be used when sensitising communities [46].

The identified barriers and the inductive themes are listed in the table below, along with sample quotes from participants.

## Discussion

This qualitative review of 17 studies offers a comprehensive exploration of the socio-cultural, emotional, and systemic barriers to cervical cancer screening in LMICs. The included evidence is geographically diverse but unevenly distributed, with approximately half of the studies conducted in sub-Saharan Africa (including Nigeria, Cameroon, Uganda, Ghana, Zimbabwe, Kenya, and Ethiopia). The remaining studies were largely from South Asia, predominantly India, with a smaller number from the Middle East, notably Iran and Iraq.

### Summary of key findings

The most prominent barriers were lack of knowledge, misconceptions, and stigma. Women were often unaware of cervical cancer screening, misunderstood its purpose, and held beliefs shaped by religious or supernatural explanations. Emotional barriers such as fear of cancer, pain, or social repercussions were prevalent, particularly where cancer was seen as a death sentence or a source of shame. Structural barriers such as cost, access to female providers, confidentiality concerns, and negative past experiences with healthcare systems further deterred women from screening.

Conversely, facilitators included community-based education, peer support, culturally sensitive interventions, and positive influence from trusted health professionals. Interventions that engaged local leaders and normalised screening within communities appeared particularly impactful.

### Comparison with previous literature

Our findings were similar to those of a scoping review by Lott et al. [23], which used an integrated behavioural model (IBM) to identify studies of interventions aimed at increasing cervical screening uptake in sub-Saharan Africa. In contrast to the review by Lott et al. [23], which did not find educational interventions to improve screening uptake, the present review identified lack of knowledge as a dominant barrier, suggesting that educational approaches may represent an important component of future implementation strategies. This is possibly due to Lott et al. [23]. including studies before 2014 and those on African women set in a non-African context, unlike the current review. Another review by Islam et al. [56]. included both qualitative and quantitative studies. In agreement with the current study, a lack of knowledge and understanding was found to be the main barrier to cancer screening programmes [57].

Consistent with prior reviews conducted among refugee and immigrant populations in High Income countries (HICs), lack of knowledge and limited understanding of cervical cancer and screening emerged as key individual-level barriers. Wearn and Shepherd [58], Haworth et al. [59], Ferdous et al. [60], and Afsah et al. [61] all reported poor cancer literacy, misconceptions about screening, and a lack of perceived need for preventive care among refugee and immigrant women. Similarly, Islam et al. [56] identified limited awareness as a dominant barrier across LMICs. However, this review extends these findings by demonstrating that, in LMIC settings, knowledge deficits are frequently compounded by culturally embedded misconceptions, including supernatural causation, fatalistic beliefs, and misunderstandings of prevention as treatment.

Emotional barriers, particularly fear and anxiety related to cancer diagnosis, death, stigma, and marital consequences, were consistently reported across the included studies. These findings closely mirror those reported by Wearn and Shepherd [58] and Afsah et al. [61], where fear of the test, fear of cancer diagnosis, and embarrassment related to genital exposure were commonly cited.

This review identified beliefs about the consequences of screening and diagnosis as a major barrier. Similar concerns regarding prognosis, pain, infertility, and disruption to family life have been reported in Wearn and Shepherd [58] and Ferdous et al. [60]. Afsah et al. [61] also documented fatalistic beliefs, including the perception of illness as divine will or punishment. Similar patterns were observed in a descriptive survey by Ubah et al. [62], where a fear of the result, lack of knowledge, lack of information and the attitudes of healthcare workers were found to be major perceived barriers, in keeping with the themes identified in this study. Shibaji et al. [63], using NFHS (National Family Health Survey) data over a five-year period in India have called for targeted mediations towards cultural taboos, reiterating the need for high-quality qualitative data.

Structural barriers, including financial constraints, transportation difficulties, and competing family responsibilities, were reported in both LMIC and HIC-based refugee studies [23, 56, 58, 61] However, unlike HIC contexts where barriers often relate to insurance coverage, appointment systems, and language services, this review highlights the importance of indirect costs and opportunity costs in LMICs, even where screening services are nominally free. Women in LMICs frequently prioritised household survival and caregiving roles over preventive care, reinforcing delays in screening uptake.

While previous reviews conducted among refugee and immigrant populations in high-income countries have provided important insights into barriers to cervical cancer screening, this review demonstrates that many of these barriers persist, and in some cases are amplified, within LMIC settings. The identification of additional themes related to femininity, supernatural beliefs, confidentiality concerns, and entrenched stigma reflects the context-specific realities of LMICs and underscores the need for interventions that are locally grounded.

### Implications for clinical practice and policy

Our results support the need for context-sensitive interventions that address both emotional and practical barriers. Health promotion campaigns should go beyond factual education to engage with local beliefs, fears, and stigmas. Involving female providers, peer educators, and trusted community figures can increase the acceptability of the programme. Importantly, addressing stigma, as rooted in sexuality, gender roles, and fear of contagion, requires broader public health messaging and system-level trust-building.

Screening models must consider the logistical challenges faced by women, such as transportation costs and childcare, and integrate screening into routine, accessible services. Interventions that provide near-home services or mobile units, as well as offer financial incentives or reduce indirect costs, may enhance uptake.

### Strengths and limitations

This study reviews the diverse qualitative perspectives across LMICs using a theory-informed analytical framework (TDF), providing an in-depth understanding of complex barriers. Although the TDF provided a useful structure for synthesis, future research could compare its utility with other behavioural or implementation frameworks in understanding screening uptake. Additionally, categorising overlapping constructs like fear-related domains remained a challenge when using the TDF; future researchers using the TDF in qualitative syntheses should anticipate construct overlap and could use dual-coding protocols early during framework mapping.

As the primary analyst, the first author's clinical background in obstetrics and gynaecology and lived experience of an LMIC may have influenced the interpretation of qualitative data. Although the TDF provided a structured analytical framework, researcher interpretation is inherent to qualitative reviews.

The scoping design allowed for a wide conceptual range and purposive sampling, which were well suited to qualitative inquiry. However, limitations include restricting the search to studies published up to 2022, which may have excluded more recent evidence. Additional limitations include the exclusion of non-English studies and specific populations (e.g., women living with HIV or disabilities), which may limit the transferability of findings. Studies were also limited to published literature post-2014, although this period coincided with an increase in cervical screening advocacy in LMICs [22, 23]. Consistent with qualitative evidence synthesis methodology, the aim was to achieve conceptual richness rather than an exhaustive historical review. Although the scoping nature precludes a formal risk of bias assessment, we adhered to Cochrane guidance to ensure rigour and transparency. Additionally, while the geographic spread of the included studies strengthens the relevance of the findings across multiple LMIC contexts, the concentration of studies in specific regions may limit transferability to under-represented settings, highlighting the need for further qualitative research in other LMIC regions.

### Future research directions

Future studies should explore the effectiveness of culturally tailored interventions, especially peer-led and community-based models. Comparative implementation research across LMIC contexts could identify scalable, adaptable solutions. Research is also needed to address gaps in understanding among marginalised groups excluded from this review, such as those with HIV or disabilities, whose barriers may differ. Furthermore, combining qualitative insights with health systems data could guide more integrated screening programmes.

## Conclusion

Women in LMICs face multifaceted barriers to cervical cancer screening, rooted in socio-cultural norms, emotional fears, and systemic challenges. Stigma, misinformation, and gender-based power dynamics remain critical obstacles. Our scoping review highlights the importance of addressing not only individual knowledge gaps but also broader societal attitudes and trust in the health system.

Facilitators such as peer support, female provider availability, and culturally appropriate education can significantly improve screening uptake. These findings point to a need for health policies that prioritise trust-building, respectful care, and social inclusion in screening initiatives.

Bridging the gaps between intervention design and real-world challenges is essential to improving cervical cancer screening uptake and ultimately reducing the global burden of cervical cancer. These findings provide a roadmap for informed, practical action in public health initiatives.

## Supplementary Information

Supplementary Material 1.

Supplementary Material 2.

Supplementary Material 3.

Supplementary Material 4.

## Data Availability

All data generated or analysed during this study are included in this published article and its supplementary materials.

## Abbreviations

HPV: Human papillomavirus
LMICs: Low- and middle-income countries
TDF: Theoretical Domains Framework
VIA: Visual inspection with acetic acid
Pap smear / PS: Papanicolaou smear
HCP: Healthcare provider
FGD: Focus Group Discussions
QIMG: Qualitative and Implementation Methods Group
SPIDER: Sample, Phenomenon of Interest, Design, Evaluation, Research type
PICO: Population, Intervention, Comparator, Outcome
WHO: World Health Organization
HICs: High-income countries
IBM: Integrated Behavioral Model
NFHS: National Family Health Survey
PPIUCD: Postpartum intrauterine contraceptive device
