# Supplementary material for: Barriers to the uptake of cervical cancer screening methods in Low- and Middle-Income Countries (LMICs) – a scoping review using framework thematic analysis
Source: BMC Womens Health. 2026 Aug 11;26:589. doi: 10.1186/s12905-026-04764-w (PMC13741580; doi:10.1186/s12905-026-04764-w)
Supplement: Supplementary file 1 [file 12905_2026_4764_MOESM1_ESM.pdf]

Appendices

Appendix 1 - Qualitative

#CONCEPTS and key words

All searched in title and abstract

#LMIC context (BOOLEAN operator – OR)

1. (Developing/ underdeveloped/ less-developed) adj5 (world/countr*)
2. Resource-limit*/resource-poor/low-resource*/resource-constrain*
3. Low-income/middle-income/lowincome/middleincome/LMIC
4. (List of 78 countries as per World Bank- lower and lower-middle income countries)

   Afghanistan* or Albania* or Algeria* or Angola* or Argentina* or Armenia* or Azerbaijan* or Bangladesh* or Belarus* or Beliz* or Benin* or Bhutan* or Bolivia* or Bosnia* or Herzegovin* or Botswan* or Brazil* or Bulgaria* or Burkina* or Burundi* or Cabo Verde* or Cape Verde* or Cambodia* or Cameroon* or Central African or Chad* or China or Chinese or Colombia* or Comor* or Congo* or Costa Rica* or Cote d'Ivoir* or Ivory Coast or Cuba* or Djibouti* or Dominica* or Ecuador* or Egypt* or El Salvador* or Eritrea* or Ethiopia* or Fiji* or Gabon* or Gambia* or Georgia* or Ghana* or Grenad* or Guatemala* or Guinea* or Guyan* or Haiti* or Hondura* or Hungar* or India* or Indonesia* or Iran* or Iraq* or Jamaica* or Jordan* or Kazakhstan* or Kenya* or Kiribati* or Korea* or Kosov* or Kyrgyz Republic or Lao* or Leban* or Lesotho* or Liberia* or Libya* or Macedonia* or Madagascar* or Malawi* or Malaysia* or Maldiv* or Mali* or Marshall Island* or Mauritania* or Mauriti* or Mexic* or Micronesia* or Moldova* or Mongolia* or Montenegr* or Morocc* or Mozambi* or Myanma* or Burmese or Namibia* or Nepal* or Nicaragua* or Niger*

or Nigeria* or Pakistan* or Palau* or Panama* or Papua New Guinea* or Paraguay* or Peru* or Philippines or Filipino or Romania* or Rwanda* or Samoa* or Sao Tome* or Senegal* or Serbia* or Seychell* or Sierra Leon* or Solomon Island* or Somalia* or South Africa* or Sudan* or Sri Lanka* or St Lucia* or St Vincent or Grenadines or Surinam* or Swazi* or Syria* or Tajikistan* or Tanzania* or Thai* or Timor* or Togo* or Tonga* or Tunisia* or Turk* or Turkmenistan* or Tuvalu* or Uganda* or Ukrain* or Uzbekistan* or Vanuatu* or Venezuela* or Vietnam* or West Bank or Gaza or Yemen* or Zambia* or Zimbabwe*

AND

(#Receivers

1. Women (MeSH)

OR

#Providers

1. Health Personnel (MeSH))

AND

(#Psychosocial Barriers and Facilitators concept

1. Social stigma/
2. Quality of life/
3. Anxiety/
4. Blame/
5. Harm/
6. Regret/
7. Embarrassment/
8. Psychologic*/

9. Emotion*/
10. Sexual behaviour/
11. Inhibiting factor*/
12. Knowledge/
13. Experience*/
14. Observation*/
15. Perception*/
16. View*/
17. Barrier*/
18. Obstacle*/
19. Facilitator*/
20. Attitude*/
21. Behavi*r/
22. Compliance/
23. Complie*/
24. Comply*/
25. Dropout*/
26. Drop out/
27. Uptake/
28. Particp*/
29. Nonattend*/
30. Accept/
31. Attend/
32. Utili*ation/

33. Refus*/
34. Reluctan*

OR

#Implementation Concept

1. Guideline Adherence(MeSH)/ Guideline (MeSH)/ Practice Guidelines as Topic(MeSH)/ Recommendation* AND
2. Barrier*/obstacle*/facilitator*/adherence)

AND

#Qualitative Concept

1. Qualitative Research (MeSH) /
2. Ethnography/
3. Qualitative/
4. Grounded-theory/
5. Thematic-analysis/
6. Content-analysis/
7. Observational-methods/
8. Constant-comparative-method/
9. Field-notes/
10. Participant-observation/
11. Narratives/
12. Field-studies/
13. Focus-groups

AND

1. Patient Education as Topic(MeSH)/

2. Health Education (MeSH)/
3. Reminder Systems(MeSH)/
4. Health Communication (MeSH)/
5. Community Health Aides (MeSH)
6. Allied Health Personnel (MeSH)
7. Community or village* NEAR/3 (intervention*/support/treatment*/visit*)
8. Encourage*/
9. Improve*/
10. Uptake/
11. Increas*/

AND

#cervical cancer concept

1. Vaginal smears/
2. Vagina* adj5 smear*
3. Pap* adj5(test* or smear)/
4. Cervi* ajd5 (smear* or screen*)/
5. Cytology/
6. Cytobrush/
7. Uterine cervical Neoplasms(MeSH)/
8. Cervical cancer/
9. Cervical neoplasm*

AND

#screening concept

10. Mass Screening(MeSH)/

11. Early Detection of Cancer(MeSH)/

12. Screening/

13. Self-sampling/

14. Self-collection/
