# Supplementary material for: Barriers to the uptake of cervical cancer screening methods in Low- and Middle-Income Countries (LMICs) – a scoping review using framework thematic analysis
Source: BMC Womens Health. 2026 Aug 11;26:589. doi: 10.1186/s12905-026-04764-w (PMC13741580; doi:10.1186/s12905-026-04764-w)
Supplement: Supplementary file 2 [file 12905_2026_4764_MOESM2_ESM.pdf]

Appendix 2

TDF domain definitions

1) Knowledge – "An awareness of the existence of something"
2) Skills – "An ability or proficiency acquired through practice"
3) Social/professional role and identity – "A coherent set of behaviours and displayed personal qualities of an individual in a social or work setting"
4) Beliefs about capabilities – "Acceptance of the truth, reality or validity about an ability, talent or facility that a person can put to constructive use"
5) Optimism – "The confidence that things will happen for the best or that desired goals will be attained"
6) Beliefs about consequences – "Acceptance of the truth, reality or validity about outcomes of a behaviour in a given situation"
7) Reinforcement – "Increasing the probability of a response by arranging a dependent relationship or contingency, between the response and a given stimulus"
8) Intentions – "A conscious decision to perform a behaviour or a resolve to act in a certain way"
9) Goals – "Mental representations of outcomes or end states that an individual wants to achieve"
10) Memory, attention, and decision processes – "The ability to retain information, focus selectively on aspects of the environment and choose between two or more alternatives"
11) Environmental context and resources – "Any circumstance of a person's situation or environment that discourages or encourages the development of skills and abilities, independence, social competence and adaptive behaviour"
12) Social influences – "Those interpersonal processes that can cause individuals to change their thoughts, feelings or behaviours"
13) Emotion – "A complex reaction pattern, involving experiential, behavioural and physiological elements, by which the individual attempts to deal with a personally significant matter or event"
14) Behavioural regulation – "Anything aimed at managing or changing objectively observed or measured actions"
