# Supplementary material for: Barriers to the uptake of cervical cancer screening methods in Low- and Middle-Income Countries (LMICs) – a scoping review using framework thematic analysis
Source: BMC Womens Health. 2026 Aug 11;26:589. doi: 10.1186/s12905-026-04764-w (PMC13741580; doi:10.1186/s12905-026-04764-w)
Supplement: Supplementary file 4 [file 12905_2026_4764_MOESM4_ESM.docx]

**Supplementary Table S1. Illustrative mapping of qualitative findings to TDF domains- Examples**

| **Illustrative qualitative finding** | **Source study** | **Descriptive code/theme** | **TDF domain(s)** | **Rationale for mapping** |
| --- | --- | --- | --- | --- |
| “None of the participants in this group previously believed that cervical cancer is becoming a significant problem or that they could get cervical cancer.” | Modibbo et al., 2016 | Lack of knowledge on cervical cancer | Knowledge | The extract directly indicates limited awareness of cervical cancer and personal susceptibility. |
| “I don’t think PS is needed for anyone who has a normal delivery.” | Marashi et al., 2021 | misconceptions; Apparent sense of health | Knowledge | The participant expresses an inaccurate understanding of who requires screening and when it is needed. |
| “I have heard of the disease on the radio, but I have not heard of the screening; that is why I have not screened.” | Binka et al., 2019 | Lack of knowledge on cervical screening | Knowledge | The extract directly indicates awareness of cervical cancer but lack of knowledge about the availability of screening. |
| “Being diagnosed with cervical cancer is like a death sentence so women prefer not to know.” | Lunsford et al., 2017 | fear of cancer; fear of abnormal results | Emotion; Beliefs about consequences | The finding contains both an emotional response (fear) and an expectation of a severe negative outcome following diagnosis. |
| Participants described needing spousal permission and preferring a female doctor because of religious and cultural expectations. | Modibbo et al., 2016 | spousal influence; religious identity | Social influences; Social and or professional role and identity | Spousal permission represents interpersonal influence, while preference for a female examiner reflects culturally and religiously shaped identity and role expectations. |
| A husband may interpret screening as evidence that his wife has been promiscuous, potentially causing marital conflict or family breakdown. | Lunsford et al., 2017 | stigma; association with sex | Emotion; Social influences | The finding reflects shame and fear experienced by women, alongside anticipated judgement and consequences imposed by partners and the wider social environment. |
| “Scared, the family members may not mingle with us casually… they will isolate us.” | Mahalakshmi and Suresh, 2020 | stigma; social outcast | Emotion; Social influences | Fear of rejection is an emotional response, while isolation by family or community represents an interpersonal and social influence on screening behaviour. |
| “For now, I am not working. If I had a job, I would have paid for the cost of the test.” | Binka et al., 2019 | financial | Reinforcement | The financial cost changes the likelihood of screening attendance by making the behaviour less feasible or rewarding. |
| “All women endorsed peer-to-peer education and engaging local leaders as some of the best strategies to recruit women.” | Teng et al., 2014 | peer support or influence | Reinforcement; Social influences | Peer encouragement and endorsement by local leaders can increase the likelihood of screening and operate through interpersonal and community influence. |
| Women preferred provider-collected samples because they believed they might be unable to obtain an adequate-quality sample themselves. | Modibbo et al., 2016 | lack of confidence in self-sampling | Skills | The finding concerns perceived practical ability to perform the sampling procedure correctly |
| Women described long queues, travel to surrounding cities and limited availability of obstetricians as barriers to screening. | Marashi et al., 2021 | convenience | Environmental context and resources | The finding concerns the practical accessibility, time and travel demands of attending screening services. |
